# β_2_-Adrenoceptor Activation Stimulates IL-6 Production via PKA, ERK1/2, Src, and Beta-Arrestin2 Signaling Pathways in Human Bronchial Epithelia

**DOI:** 10.1007/s00408-021-00484-0

**Published:** 2021-11-01

**Authors:** Rui-Gang Zhang, Ya Niu, Ke-Wu Pan, Hao Pang, Chun-Ling Chen, Chung-Yin Yip, Wing-Hung Ko

**Affiliations:** 1 Department of Physiology, Basic Medical School, Guangdong Medical University, Zhanjiang, China; 2 School of Biomedical Sciences, The Chinese University of Hong Kong, Hong Kong, N.T. China

**Keywords:** β_2_-Adrenoceptor, IL-6, Bronchial epithelia, PKA, ERK1/2, β-Arrestin2

## Abstract

**Objective:**

β_2_-Adrenoceptor agonists are widely used to treat asthma because of their bronchial-dilation effects. We previously reported that isoprenaline, via the apical and basolateral β_2_-adrenoceptor, induced Cl^−^ secretion by activating cyclic AMP (cAMP)-dependent pathways in human bronchial epithelia. Despite these results, whether and how the β_2_-adrenoceptor-mediated cAMP-dependent pathway contributes to pro-inflammatory cytokine release in human bronchial epithelia remains poorly understood.

**Methods:**

We investigated β_2_-adrenoceptor-mediated signaling pathways involved in the production of two pro-inflammatory cytokines, interleukin (IL)-6 and IL-8, in 16HBE14o- human bronchial epithelia. The effects of isoprenaline or formoterol were assessed in the presence of protein kinase A (PKA), exchange protein directly activated by cAMP (EPAC), Src, and extracellular signal-regulated protein kinase (ERK)1/2 inhibitors. The involvement of β-arrestin2 was examined using siRNA knockdown.

**Results:**

Isoprenaline and formoterol (both β_2_ agonists) induced IL-6, but not IL-8, release, which could be inhibited by ICI 118,551 (β_2_ antagonist). The PKA-specific inhibitor, H89, partially inhibited IL-6 release. Another intracellular cAMP receptor, EPAC, was not involved in IL-6 release. Isoprenaline-mediated IL-6 secretion was attenuated by dasatinib, a Src inhibitor, and PD98059, an ERK1/2 inhibitor. Isoprenaline treatment also led to ERK1/2 phosphorylation. In addition, knockdown of β-arrestin2 by siRNA specifically suppressed cytokine release when a high concentration of isoprenaline (1 mM) was used.

**Conclusion:**

Our results suggest that activation of the β_2_-adrenoceptor in 16HBE14o- cells stimulated the PKA/Src/ERK1/2 and/or β-arrestin2 signaling pathways, leading to IL-6 release. Therefore, our data reveal that β_2_-adrenoceptor signaling plays a role in the immune regulation of human airway epithelia.

## Introduction

β_2_-Adrenoceptor agonists are the most widely used and effective therapies for ameliorating asthmatic symptoms. However, clinical application of β_2_-adrenoceptor agonists has been shown to enhance airway inflammation [1], triggering a paradox in asthma management [2, 3]. During the inhalation of β_2_-adrenoceptor agonists, airway epithelial cells are exposed to the drug, leading to potential side effects that may also require attention. It has been reported that β_2_-adrenoceptor activation in the airway epithelium can restore and promote the cardinal features of asthma, including eosinophilic inflammation, mucous metaplasia, and airway hyperresponsiveness [4]. Therefore, although β_2_-adrenoceptor agonists are expected to dilate airway smooth muscle, their activation of epithelial β_2_-adrenoceptors may play a major role in causing inflammation in patients taking these therapeutics, which could in turn lead to further asthma symptoms.

β_2_-Adrenoceptor agonists are known to activate cyclic AMP (cAMP)-dependent kinase signaling pathways [5, 6]. We recently reported that isoprenaline stimulated β_2_-adrenoceptors and induced Cl^−^ secretion, which was mediated through the cAMP-dependent protein kinase A (PKA) pathway, leading to the activation of cystic fibrosis transmembrane conductance regulator (CFTR) in human bronchial epithelia [7]. However, whether the cAMP/PKA signaling pathway is involved in aggravating inflammation by promoting secretion of pro-inflammatory cytokines remains largely unknown. Apart from PKA, exchange protein directly activated by cAMP (EPAC) has been found to be a novel cAMP-responding downstream molecule associated with many biological functions [8].

Moreover, studies have shown that β_2_-adrenoceptor agonists activate G protein-independent pathways mediated by β-arrestins [9], which were initially expected to mediate receptor internalization and desensitization [10]. The latest findings indicate that β-arrestins, acting as scaffolds to recruit signaling molecules, are capable of activating Src family non-receptor tyrosine kinases and mitogen-activated protein kinases (MAPKs), which then proceed to activate pro-inflammatory cytokine release [11, 12].

In this study, we examined the involvement of both PKA and EPAC in β_2_-adrenoceptor activation to expand our knowledge of cAMP-dependent pathways in asthma. The effects of common pro-inflammatory pathways, such as Src and extracellular signal-regulated protein kinase (ERK)1/2, were also examined in our study. We characterized the release of interleukin (IL)-6 and IL-8 induced by various concentrations of a β_2_-adrenoceptor agonist, isoprenaline, and a more specific β_2_-adrenoceptor agonist, formoterol. Our data demonstrate that β_2_-adrenoceptor activation led to IL-6, but not IL-8, production. PKA/Src, as well as ERK1/2, activated IL-6 release when relatively low concentrations of isoprenaline were used. However, β-arrestin2 was involved in IL-6 expression following treatment with high concentrations of isoprenaline. These findings expand our knowledge of the important role β_2_-adrenoceptor agonists play in human airway inflammation and thus have clinical implications for future asthma research and therapy.

## Materials and Methods

### Reagents

All pharmacological tools were obtained from Sigma-Aldrich (St Louis, MO, USA). All cell culture reagents were obtained from Invitrogen (Carlsbad, CA, USA), unless otherwise stated.

### Cell Culture

The human 16HBE14o- bronchial epithelia cell line was cultured and maintained as described previously [13]. After cells reached confluence, they were placed in serum-free conditions overnight before drug delivery and sample collection. For experiments, cells were grown in 24-well culture plates [14].

### ELISA

Cells were grown in 24-well culture plates and cell-free supernatants were collected from control and treated cells before analysis using a commercially available ELISA kit specific for IL-6 (Invitrogen eBioscience, Carlsbad, CA, USA) and IL-8 (BD Biosciences, San Diego, CA, USA) according to the manufacturers’ protocols. All experiments were performed in duplicate.

### Small Interference RNA Transfection

β-arrestin2 knockdown by small interference RNA (siRNA) was performed as described previously [14]. Briefly, the medium was changed to OPTI-MEM™ I Reduced Serum Medium (Gibco, Invitrogen, USA) after cells reached 60%–70% confluence. Transfection of 25 nM β-arrestin2 siRNA (Thermo Fisher Scientific, Waltham, MA, USA, Cat.: AM16708) or control siRNA (Thermo Fisher Scientific, Cat.: AM4613) was achieved using Lipofectamine® RNAiMAX Reagent (Invitrogen) 24 h before isoprenaline was added.

### Western Blotting

SDS-PAGE (Bio-Rad Laboratories, Hercules, CA, USA) followed by transfer to polyvinylidene fluoride membranes (Immobilon-P, Millipore Corporation, Billerica, MA, USA) was performed. Anti-phospho-p44/42 MAPK (ERK 1/2; Cell Signaling Technology; Danvers, MA; 1:1,000) and anti-p44/42 MAPK (ERK 1/2; Cell Signaling Technology; 1:1,000) primary antibodies were used.

### Real-Time PCR

Total RNA was extracted using TRIzol™ Reagent and reverse transcribed into cDNA using iScript™ Reverse Transcription Supermix (Bio-Rad Laboratories). Real-time PCR was conducted using the Applied Biosystems Power SYBR Green PCR Master Mix in an ABI QuantStudio1 thermal cycler (Applied Biosystems, Life Technologies, Carlsbad, CA, USA). The following primer sequences were used (5’-3’): *gapdh* forward, CGGGAAGGAAATGAATGGGC; reverse, GCCCAATACGACCAAATCAGAGAAT; *beta-arrestin2* forward, GGAAGCTGGGCCAGCAT; reverse, TGTGACGGAGCATGGAAGATT.

### Statistical Analysis

Data are expressed as the means ± standard error (S.E.). Comparisons between control and treated epithelia were performed using the Student’s *t* test or one-way analysis of variance (ANOVA) with Dunnett’s post hoc test (GraphPad Prism 5) as appropriate.

## Results

### ***β***_2_-Adrenoceptor is Involved in Isoprenaline-Induced Secretion of IL-6 But Not IL-8

**Fig. 1:**
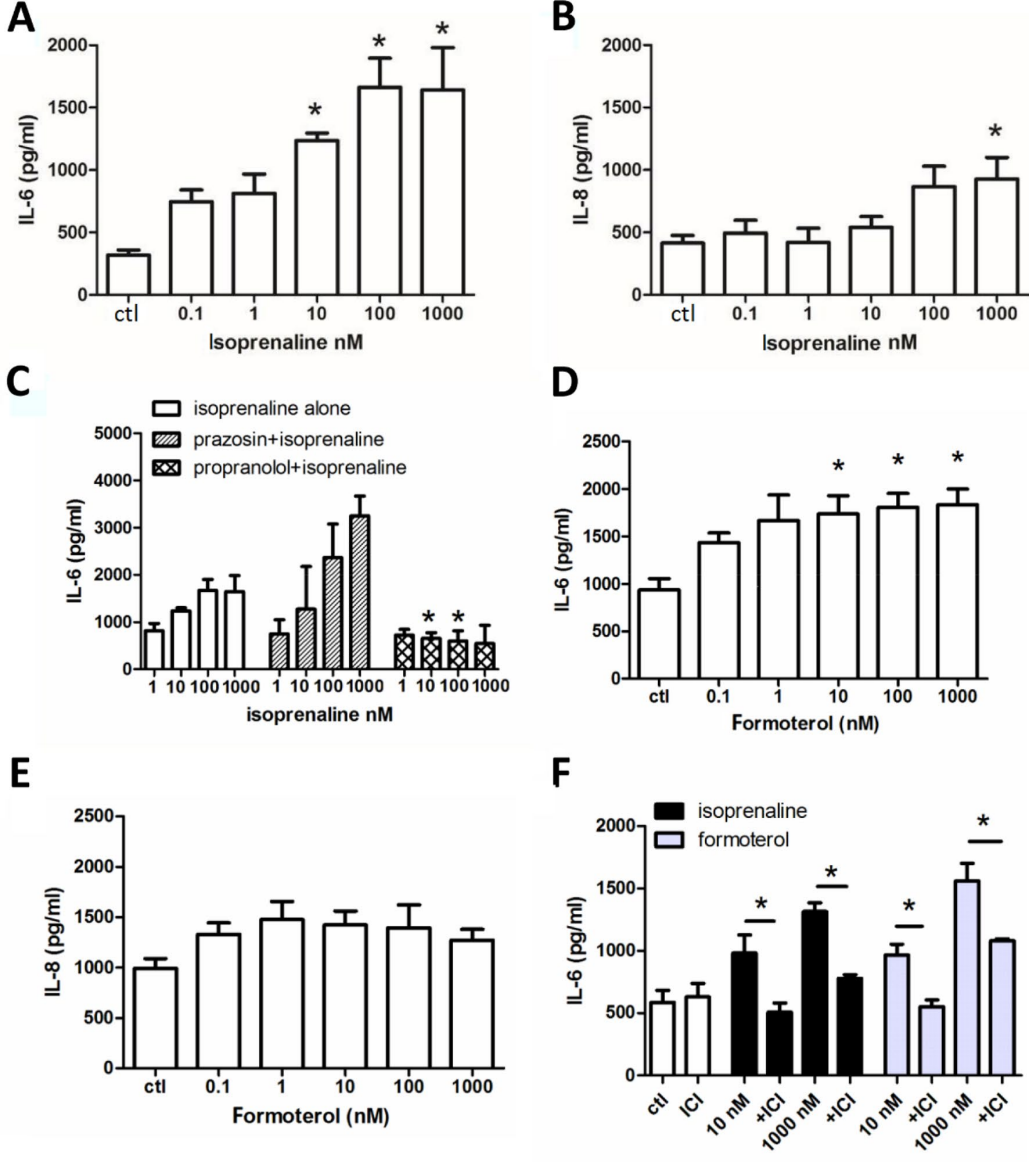
Isoprenaline or formoterol induce IL-6 and IL-8 release. **A**–**B** 16HBE14o- cells were treated with different concentrations of isoprenaline for 6 h. IL-6 (**A**) and IL-8 (**B**) release were quantified by ELISA (*n* = 5–7). **C** Cells were pretreated with prazosin (1 μM) or propranolol (10 μM) for 2 h, and then the cells were stimulated with isoprenaline in the presence of the inhibitors prior to quantification of IL-6 secretion by ELISA (*n* = 4–6). **D**–**E** 16HBE14o- cells were treated with different concentrations of formoterol for 6 h and then IL-6 (**D**) and IL-8 (**E**) release were quantified by ELISA (*n* = 4–5). **F** Cells were pretreated with ICI 118,551 (10 μM; ICI) for 2 h before stimulation of the cells with isoprenaline or formoterol for 6 h (*n* = 3–5). Each column represents the mean ± S.E. (For A, B, D & E: *p < 0.05 compared with the control (ctl) group; one-way ANOVA with Dunnett’s post hoc test; For C & F: *p < 0.05 compared with the same concentration of isoprenaline or formoterol without inhibitor; Student's t-test)

We previously demonstrated that isoprenaline activates β_2_-adrenoceptor in 16HBE14o- cells to evoke Cl^−^ secretion via a cAMP-dependent pathway [7]. Here, we examined the involvement of β_2_-adrenoceptors using pharmacological tools and quantified isoprenaline-induced IL-6 and IL-8 production by ELISA. Stimulation of 16HBE14o- cells with different concentrations of isoprenaline (0.1 nM – 1 μM) for 6 h caused an increase in IL-6 secretion in a concentration-dependent manner (Fig. 1A). Compared to IL-6 secretion, the same concentrations of isoprenaline did not cause a significant increase in IL-8, except at the highest concentration of 1 μM (Fig. 1B). The specific α1-receptor antagonist prazosin and the β-receptor antagonist propranolol were employed to verify the involvement of α-and β-adrenoceptors in IL-6 secretion (Fig. 1C). Cells were pretreated with prazosin (1 μM) or propranolol (10 μM) for 2 h and then stimulated with isoprenaline in the presence of the antagonists for 6 h. Figure 1C shows that propranolol (10 μM) but not prazosin (1 μM) inhibited isoprenaline-induced IL-6 release when compared to the respective controls without inhibitor. To further demonstrate the involvement of the β_2_-adrenoceptor, we examined the effect of formoterol, a specific β_2_-adrenoceptor agonist, and ICI 118,551, a specific β_2_-adrenoceptor antagonist, on IL-6 and IL-8 secretion. Similar to the effects of isoprenaline, formoterol-induced IL-6 (Fig. 1D) but not IL-8 (Fig. 1E) release in a concentration-dependent manner. The effects of both isoprenaline and formoterol on IL-6 production were significantly blocked by ICI 118,551 (Fig. 1F). Taken together, these results confirmed that isoprenaline could induce IL-6 secretion by activating β_2_-adrenoceptor. Because isoprenaline and formoterol did not significantly increase IL-8 secretion, the inhibitory effects of prazosin, propranolol, and ICI 118,551 on isoprenaline- or formoterol-induced cytokine secretion were not investigated further.

### cAMP-Dependent Signaling Pathways Mediate Isoprenaline-Induced IL-6 Release

**Fig. 2:**
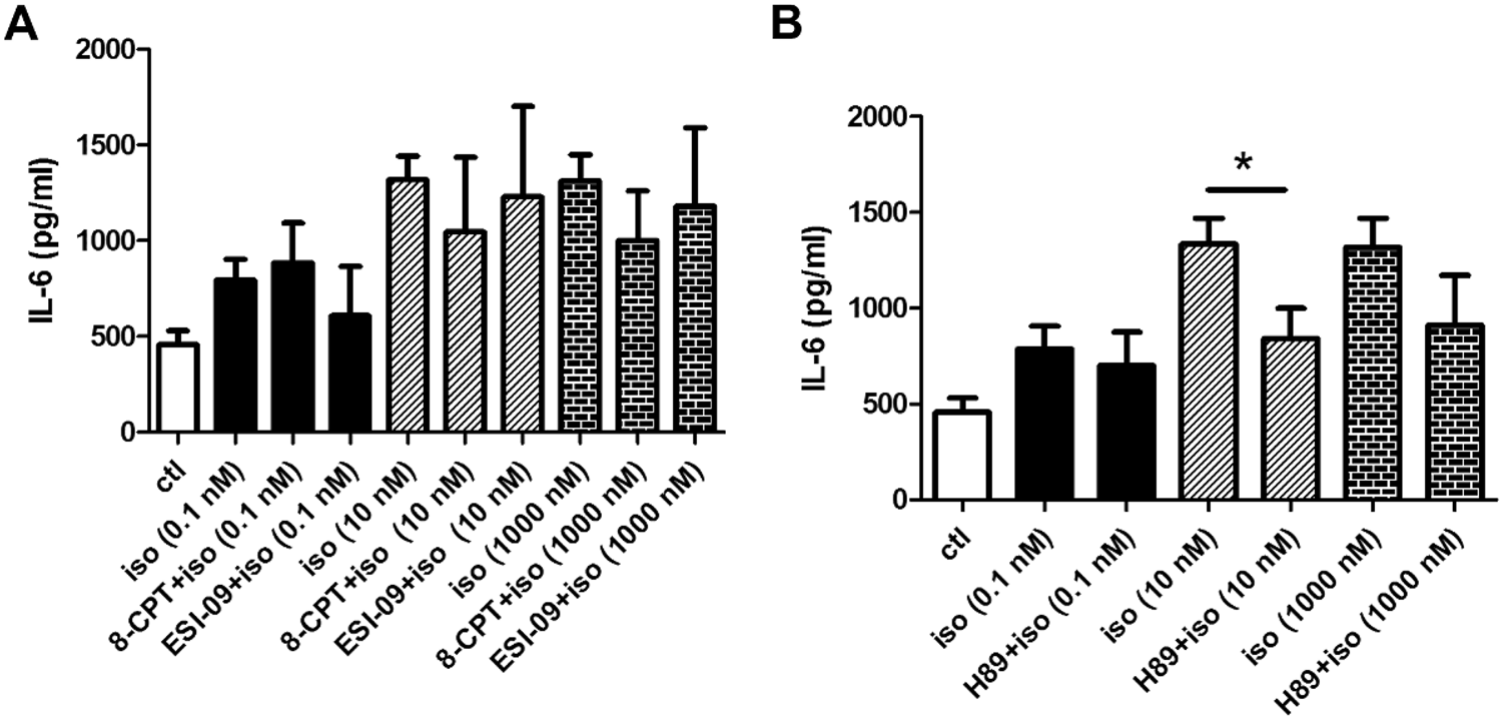
cAMP-dependent signaling pathways are involved in isoprenaline-mediated IL-6 release. **A** Cells were pretreated with EPAC activator 8-CPT (5 μM) or EPAC inhibitor ESI-09 (5 μM) for 2 h before addition of isoprenaline (iso) for 6 h. IL-6 was quantified by ELISA. **B** The effect of PKA inhibitor H89 (10 μM) on isoprenaline-induced IL-6 was examined. Each column represents the mean ± S.E. *n* = 3–5; *p < 0.05, as calculated by the Student's t-test

We previously showed that isoprenaline could activate β_2_-adrenoceptor-dependent signaling pathways in 16HBE14o- cells, leading to an increase in intracellular cAMP level [7]. In this study, we investigated the involvement of two intracellular receptors of cAMP, namely PKA and EPAC, on IL-6 secretion. The effect of EPAC activation was examined by using an analog of cAMP (8-pCPT-2’-O-Me-cAMP; 8-CPT) which specifically activates EPAC protein [15]. We also used ESI-09, a novel non-cyclic nucleotide EPAC antagonist [16]. Compared to control samples, pretreatment of the epithelial cells with 5 µM 8-CPT or ESI-09 for 2 h before stimulating the cells with different concentrations of isoprenaline did not affect IL-6 secretion (Fig. 2A), suggesting that EPAC protein is not involved in isoprenaline-induced IL-6 secretion. However, treatment with 10 μM H89, a PKA-specific inhibitor, significantly suppressed isoprenaline-induced IL-6 release (Fig. 2B). Activation of adenylyl cyclase (AC) by forskolin has been reported to elevate intracellular cAMP level in 16HBE14o- cells [17], presumably leading to activation of PKA and EPAC. Stimulating the cells with 1000 nM isoprenaline for 6 h increased the IL-6 secretion (1313.7 ± 137.4 pg/ml; *n* = 10). This increase was not statistically different from treatment with isoprenaline and 5 µM forskolin (1130.0 ± 449.1 pg/ml; *n* = 4; *p* > 0.05 *vs* isoprenaline only; Student’s *t* test) or isoprenaline together with 5 µM forskolin and ESI-09 (1465.4 ± 160.1 pg/ml; *n* = 6; *p* > 0.05 *vs* isoprenaline only; Student’s *t* test). Therefore, no synergistic increase in IL-6 secretion was observed when epithelial cells were treated with both a β_2_-adrenoceptor agonist and cAMP-mobilizing agent. Similar results were obtained when the cells were treated with 10 nM isoprenaline (data not shown). Taken together, these results indicate that the IL-6 secretion stimulated by β_2_-adrenoceptor activation utilizes the same signaling pathway induced by forskolin treatment, namely the AC/cAMP signaling cascade followed by PKA but not EPAC activation.

### Involvement of Src and ERK1/2 Signaling Pathways in Isoprenaline-Induced IL-6 Production

**Fig. 3:**
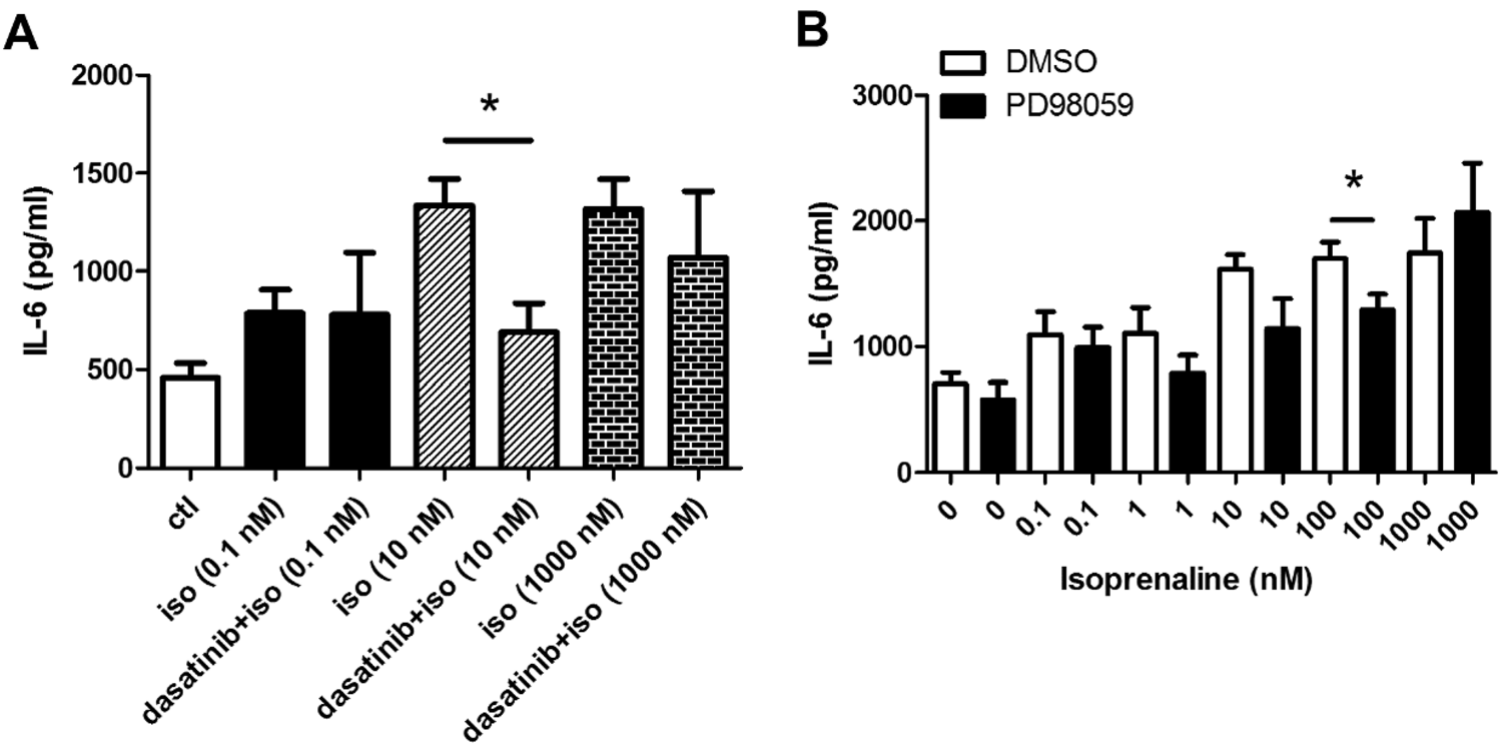
Src and ERK1/2 are involved in isoprenaline-induced IL-6 secretion. **A**–**B** Cells were treated with dasatinib (10 μM) or PD98059 (10 μM) for 2 h before isoprenaline (iso) treatment at different concentrations for 6 h. The effect of dasatinib (**A**) and PD98059 (**B**) on IL-6 release was examined. Each column represents the mean ± S.E. *n* = 4–6; *p < 0.05, as calculated by the Student's t-test

A recent study suggested that Src-mediated MAPK signaling pathways are involved in kidney inflammation [18]. To delineate whether Src, a typical family of non-receptor tyrosine kinases, is also involved in isoprenaline-induced IL-6 secretion, 16HBE14o- cells were stimulated with different concentrations of isoprenaline in the presence or absence of dasatinib, a Src inhibitor [19]. Figure 3A shows that treatment with 10 μM dasatinib significantly suppressed 10 nM isoprenaline-induced IL-6 secretion.

**Fig. 4:**
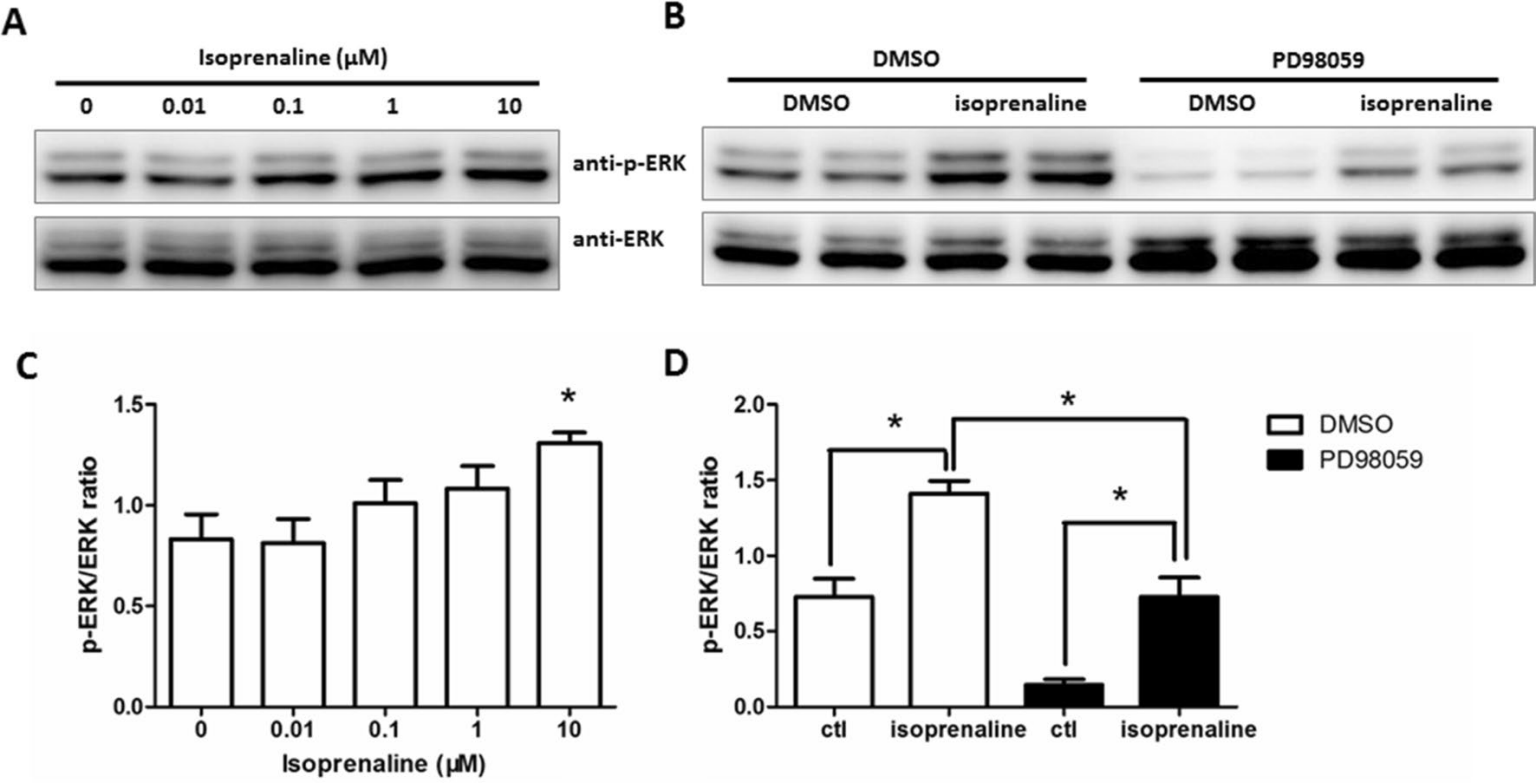
Effect of isoprenaline on ERK1/2 phosphorylation. **A** 16HBE14o- cells were stimulated with different concentrations of isoprenaline for 5 min. **B** Cells were treated with DMSO or PD98059 (10 μM) for 15 min followed by isoprenaline (10 μM) stimulation for 5 min. Representative images of western blots are shown. *n* = 3. Summarized data are shown in **C** and **D,** showing the quantification of p-ERK levels normalized to total ERK (*p < 0.05, as calculated by the Student's t-test or ANOVA as appropriate)

ERK1/2 is an important pro-inflammatory cytokine release mediator among different MAPKs in human airway epithelial cells [20]. Treatment of the cells with an ERK1/2 inhibitor, PD98059 (10 μM), reduced the level of IL-6 release induced by isoprenaline (Fig. 3B). Examination of ERK1/2 phosphorylation status by western blot analysis revealed that different concentrations of isoprenaline can induce ERK1/2 phosphorylation (Fig. 4A, C). Moreover, PD98059 significantly inhibited basal (DMSO) and isoprenaline-induced ERK1/2 phosphorylation (Fig. 4B, D). These data suggest that the Src and ERK1/2 signaling pathways are involved in isoprenaline-induced IL-6 secretion.

### β-Arrestin2 Plays a Role in Isoprenaline-Induced IL-6 Production

**Fig. 5:**
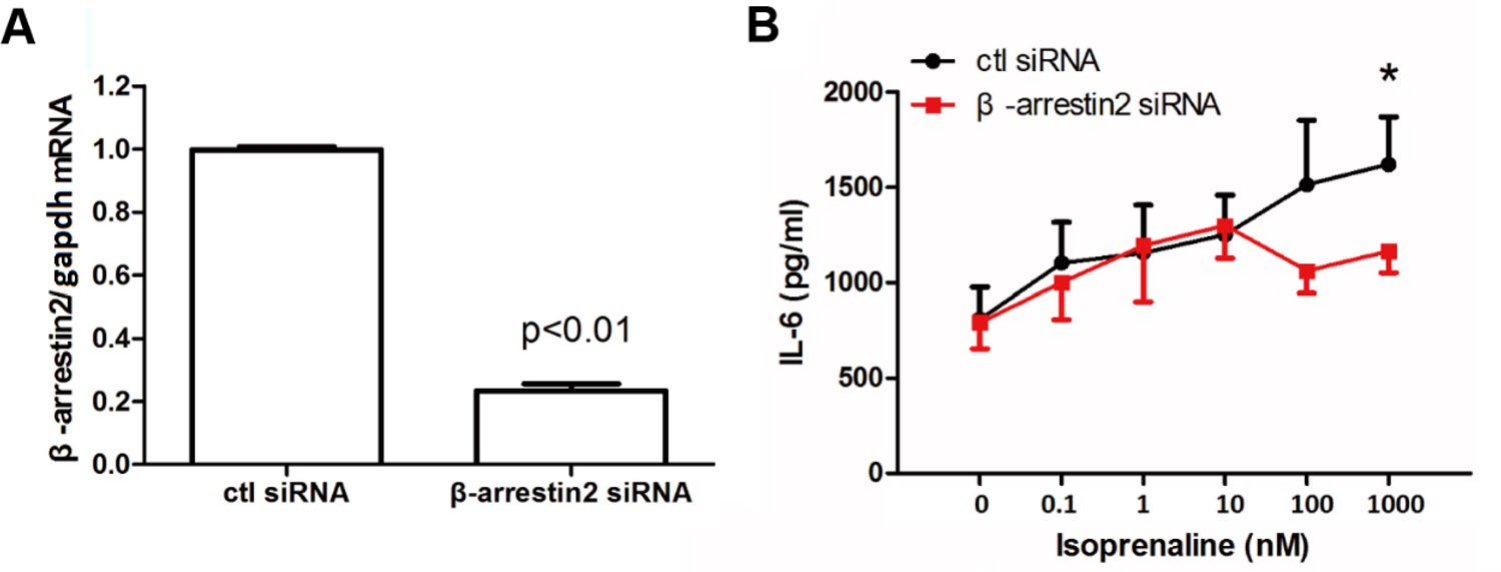
β-Arrestin2 mediates isoprenaline-induced IL-6 release. **A** The efficiency of β-arrestin2 knockdown (KD) was verified by real-time PCR (*n* = 3). The expression of β-*arrestin2* mRNA was normalized by the level of *GAPDH* mRNA. **B** The effect of β-arrestin2 KD on isoprenaline-induced IL-6 release was examined. Each data point represents the mean ± S.E. *n* = 5; *p < 0.05 compared with the same concentration of isoprenaline between the control (ctl siRNA) and KD groups (β-arrestin2 siRNA), as calculated by the Student’s t-test

β-arrestin2 is involved in Src and MAPK signaling pathways and inflammatory responses [12, 21]. To examine the involvement of β-arrestin2 in isoprenaline-induced IL-6 secretion, we depleted β-arrestin2 mRNA by siRNA (Fig. 5A) and studied the effect of this knockdown on IL-6 production. β-arrestin2 knockdown led to a significant decrease in IL-6 secretion induced by a relatively high concentration of isoprenaline (1 μM) (Fig. 5B).

## Discussion

In the present study, we found that isoprenaline-induced IL-6, but not IL-8, production via β_2_-adrenoceptor-mediated signaling pathways. After activation of AC/cAMP signaling, PKA, but not EPAC, played a role in isoprenaline-mediated IL-6 secretion. In addition, both Src- and ERK1/2-dependent signaling pathways were involved. β-Arrestin2, however, was only involved in mediating isoprenaline-induced IL-6 production at relatively high concentrations. Thus, these results indicate that β_2_-adrenoceptor/AC/cAMP/PKA, Src and ERK1/2 are involved in mediating IL-6 release in 16HBE14o- cells. IL-6 and IL-8 are the two pro-inflammatory cytokine markers secreted by human airway epithelia during inflammation [20, 22]. These two classic proinflammatory cytokines are implicated in the initiation and perpetuation of local airway inflammatory responses and play important roles in bronchial epithelial cells [23, 24]. Our data suggest that activation of β_2_-adrenoceptor is only associated with IL-6, but not IL-8, secretion. IL-6 is elevated in asthmatic patients [25] and it is suggested that IL-6 has a causative role in determining an increase in airway resistance in asthma and chronic obstructive pulmonary disease [26].

PKA and EPAC are the two major downstream targets of cAMP [8]. Since β_2_-adrenoceptors are known to activate the AC/cAMP-dependent pathway in 16HBE14o- cells to activate CFTR, we examined the role of PKA and EPAC in isoprenaline-induced cytokine release. EPAC has been reported to take part in mediating inflammation [27] and activating ERK1/2 [28]. PKA and EPAC either cooperate [29, 30] or act independently [31] to regulate many biological functions. Although EPAC protein is expressed in 16HBE14o- cells [32], the results in Fig. 2 suggest that PKA, but not EPAC, participates in isoprenaline-induced IL-6 release. Our results are in contrast with other reports which suggest that the β_2_-adrenoceptor is associated with PKA-independent factors, such as EPAC. β_2_-adrenoceptor agonist induced IL-1β and IL-6 production through PKA-independent mechanisms in a murine macrophage cell line [33]. Activation of β_2_-adrenoceptors induced IL-6 production in neonatal mouse cardiac fibroblasts through an AC/cAMP/p38 MAPK pathway that was independent of PKA [34]. One possibility is that the involvement of EPAC in cytokine release appears to be cell-type-specific and may be related to pathophysiology during inflammation and specific innate immune protection in different tissues. Another possibility is that the aforementioned studies employed such high concentrations of β_2_-adrenoceptor agonist (e.g., 0.5 μM salmeterol; 10 μM isoprenaline) that cAMP-independent pathways such as β-arrestin2 become activated (Fig. 5). To the best of our knowledge, we are the first to study the effects of β_2_-adrenoceptor agonists across a wide range of concentrations, including relatively low concentrations (from 0.1 nM to 1 μM), on cytokine release.

Similar to PKA (Fig. 2B), the Src family tyrosine kinase inhibited IL-6 secretion (Fig. 3). Dasatinib is an FDA-approved small-molecule drug used to treat myeloid leukemia [35] that potently inhibits Src family kinase [36]. PKA is reported to be able to activate Src signaling by phosphorylating the kinase at residue serine 17 [37, 38]. Whether PKA suppresses IL-6 secretion through Src remains to be studied. It has been widely reported that β_2_-adrenoceptor agonist-mediated cytokine release occurs through ERK1/2 and/or p38 MAPK [9, 33, 34]. Our study is consistent with these reports as we demonstrate that PD98059 not only inhibited ERK1/2 phosphorylation (Fig. 4) but also suppressed IL-6 secretion (Fig. 3B).

β-Arrestin2 knockdown specifically inhibited the effect of high isoprenaline concentrations (Fig. 5). G protein-coupled receptor kinases (GRKs) typically mediate β-adrenoceptor phosphorylation followed by β-arrestin recruitment. β-arrestins act as a scaffold to further activate other signaling molecules, such as ERK1/2 [12, 39]. Our results strongly suggest that β-arrestin2 mediates β_2_-adrenoceptor agonist (e.g., isoprenaline)-induced cytokine release. This may be the case when patients are exposed to a relatively high concentration of agonists. Apart from IL-6, we observed an inhibitory effect of β-arrestin2 knockdown on IL-8 secretion induced by 1 µM isoprenaline (data not shown). Therefore, it is possible that GRK may phosphorylate the β_2_-adrenoceptor upon stimulation and induce β-arrestin2 recruitment to inhibit cytokine release via ERK1/2. The cAMP pathway has been shown to activate either p38 MAPK [40] or ERK1/2 [41]. Src can also activate ERK1/2 [42] and could be activated by PKA [38]. Whether Src and ERK1/2 signaling pathways are downstream of PKA has yet to be determined and awaits further investigation. Delineating the relationships between these signals would provide additional insights into the molecular events induced by β_2_-adrenoceptor activation.

Evidence that β_2_-adrenoceptor agonists are a double-edged sword in terms of asthma treatment is accumulating. Activation of cAMP/PKA pathways leads to the beneficial effects of bronchial dilation, while the involvement of β-arrestin pathways may lead to undesirable side effects, such as inflammation aggravation [11]. Bronchodilators, such as β_2_ agonists, are essential medicines used to manage symptomatic asthma. To induce bronchodilation, repeated use or increasing doses of β_2_-adrenoceptor agonist inevitably cause the airway epithelial cells to be exposed to the drug. In fact, the airway epithelial cells are the first physiochemical barrier to encounter the β_2_ agonist. Our results show how the use of β_2_-adrenoceptor drugs might affect the airway epithelia by releasing the pro-inflammatory cytokine IL-6. Further studies are required to identify other possible pro-inflammatory cytokines that are secreted upon β_2_ adrenoceptor activation in human airway epithelia.

## Conclusion

The present study demonstrates that treatment with isoprenaline (0.1 nM–1 μM) induces IL-6, but not IL-8, secretion via the β_2_-adrenoceptor in human bronchial epithelia. Several signaling pathways, including PKA, Src, and ERK1/2, are involved. High concentrations of isoprenaline (1 μM) also activate the β-arrestin2 pathway. Our findings enhance our understanding of β_2_-adrenoceptor-mediated signaling pathways and their relationship with IL-6 release in human bronchial epithelia. While activation of the β_2_-adrenoceptor in smooth muscle cells could have a bronchodilatory therapeutic effect, this is counteracted by secretion of the pro-inflammatory cytokine IL-6 by the bronchial epithelium. Thus, the paradoxical effects of β_2_-adrenoceptor activation could hamper wide spread clinical use of β_2_-adrenoceptor agonists in treating asthma.

## Abbreviations

AC: Adenylyl cyclase
CFTR: Cystic fibrosis transmembrane conductance regulator
AMP: Adenosine monophosphate
8-CPT-cAMP: 8-(4-Chlorophenylthio)adenosine-3′,5′-cyclic Monophosphorothioate
cAMP: Cyclic AMP
EPAC: Exchange protein directly activated by cAMP
ERK1/2: Extracellular signal‑regulated protein kinase
GRK: G protein-coupled receptor kinase
hrs: Hours
IL-6: Interleukin-6
IL-8: Interleukin-8
MAPK: Mitogen-activated protein kinase
PKA: Protein kinase A
